# An outer approximation method for the road network design problem

**DOI:** 10.1371/journal.pone.0192454

**Published:** 2018-03-28

**Authors:** Saeed Asadi Bagloee, Majid Sarvi

**Affiliations:** Smart Cities Transport Group, Department of Infrastructure Engineering, Melbourne School of Engineering, The University of Melbourne, Victoria, Melbourne, Australia; Southwest University, CHINA

## Abstract

Best investment in the road infrastructure or the network design is perceived as a fundamental and benchmark problem in transportation. Given a set of candidate road projects with associated costs, finding the best subset with respect to a limited budget is known as a bilevel Discrete Network Design Problem (DNDP) of NP-hard computationally complexity. We engage with the complexity with a hybrid exact-heuristic methodology based on a two-stage relaxation as follows: (i) the bilevel feature is relaxed to a single-level problem by taking the network performance function of the upper level into the user equilibrium traffic assignment problem (UE-TAP) in the lower level as a constraint. It results in a mixed-integer nonlinear programming (MINLP) problem which is then solved using the Outer Approximation (OA) algorithm (ii) we further relax the multi-commodity UE-TAP to a single-commodity MILP problem, that is, the multiple OD pairs are aggregated to a single OD pair. This methodology has two main advantages: (i) the method is proven to be highly efficient to solve the DNDP for a large-sized network of Winnipeg, Canada. The results suggest that within a limited number of iterations (as termination criterion), global optimum solutions are quickly reached in most of the cases; otherwise, good solutions (close to global optimum solutions) are found in early iterations. Comparative analysis of the networks of Gao and Sioux-Falls shows that for such a non-exact method the global optimum solutions are found in fewer iterations than those found in some analytically exact algorithms in the literature. (ii) Integration of the objective function among the constraints provides a commensurate capability to tackle the multi-objective (or multi-criteria) DNDP as well.

## Introduction

Traffic congestion is a major challenge in many cities. In addition to demand management, in many cases investment into the expansion of the road network is inevitable. Such investments should be efficient or optimum which is manifested in the well-known network design problem (NDP). The NDP by itself is regarded as a fundamental and benchmark problem in transportation science as well as operational research which has wide implications on other state-of-the-art technologies, planning, and practices.

The NDP could be classified into strategic (road constructions), tactical (road orientations, priority lanes), and operational (toll setting and signal setting). With respects to the nature of decisions variables, the NDP is also classified into the following categories: (i) continuous network design problem (CNDP), the decision variables are continuous such as signal timing setting, toll setting, and capacity expansion [1] (ii) discrete network design problem (DNDP) the decision variables are integer such as which roads must be constructed or widen and (iii) mixed network design problem (MNDP) that is a combination of continuous and integer variables [2]. This article is dedicated to the DNDP which is articulated as follows: given a limited budget and a set of projects, the best choice of affordable projects is sought. The decision variables are binary (1: build or 0: not to build the project), embedded in a bilevel programming structure: in the upper level, the non-linear objective function is minimized while solving user equilibrium traffic assignment problem (UE-TAP) in the lower level. Being bilevel -even with all objective functions and constraints being linear—is enough to make a problem NP-hard [3], that is, as the network becomes larger, the problem becomes computationally prohibitive. The discreteness of the solution space adds to the complexity of the problem.

The DNDP’s solution approaches in the literature can be viewed as exact and heuristic. The exact methods aim at the global optimum solution but due to the combinatorial trait of the problem as well as being NP-hard, their applications to real networks are restricted. The heuristic methods look for good solutions within acceptable computational time by relaxing some difficult facets of the problem. Examples are: relaxing the UE traffic flow to a system optimal [4, 5], replacing the objective function with some less expensive-to-evaluate (surrogate) function [6] employing metaheuristic algorithm such as genetic algorithm, ant system, simulated annealing [7–9], biologically inspired model [10, 11]. A thorough discussion of this can be found in [12–15]. Furthermore, in recent years, rapid expansions of developing and emerging economies (largely in Asia and the Middle East) have made the DNDP relevant more than ever [16]. Despite being time-effective, some heuristic methods are yet to appeal to the practitioners due mainly to the fact that a clear majority of such methods are essentially evolutionary based methods with random elements which result in randomly generated solutions. Considering such shortcomings in some heuristic methods, there has been a surge of interests in exact methods owing to continuous enhancements in computational technology and optimization methods. In one estimate during the course of a decade, linear optimization (which is the building-block of a variety of optimization problems) has become a million times faster [17]. Furthermore, although the size of the networks being dealt with by practitioners is large, the number of candidate projects (decision variables) is limited (say a dozen or so). Nevertheless, no matter how effective or efficient an exact method could be, facing with NP-hard problems (such as the DNDP) eventually invoke some heuristic component if the intention is to address a real-life case-study.

Given the above-mentioned properties and available computational technology as well as the state-of-the-art in optimization, in this study, we bridge the gap between exact and heuristic methods and develop a hybrid exact-heuristic method subject to two relaxation methods tailored to large-sized networks. In other words, the proposed method is basically a cross-breed method consisting of an exact method and some heuristic relaxation techniques that is to exploit the advantages of both methods to arrive at an efficient algorithm for practical uses. However, there will not be any random element in the proposed methodology as is the case in some meta-heuristic algorithms.

The discreteness and bilevel nature of the problem are the two main sources of the complexity. To crunch the complexity of the problem, a two-stage relaxation method is developed (i) the bilevel feature is relaxed to a single-level problem by taking the network performance function of the upper level into the UE-TAP in the lower level as a constraint. It results in a mixed-integer nonlinear programming (MINLP) problem which is then solved using an Outer Approximation (OA) algorithm (ii) To arrive at a tractable mixed integer linear programming (MILP) problem, we further relax the multi-commodity UE-TAP to a single-commodity MILP problem, during which, a new binary solution is sought to be evaluated (i.e. solving its corresponding UE-TAP). In other words, the intractable multi-commodity MILP is decomposed to a tractable single-commodity MILP and a tractable nonlinear UE-TAP. This methodology has two main advantages: (i) the method is proven to be highly efficient to solve the DNDP for large-size networks. The large-size network of Winnipeg, Canada consisting of 20 binary variables under various budget levels is tested. The results suggest that for such a hybrid-heuristic method, within a pre-specified maximum-iterations (as a termination criterion) global optimum solutions are quickly reached in the most of the budget levels; otherwise, good solutions (close to global optimum solutions) are found in early iterations. Comparative analysis on the networks of Gao’s network [18] and Sioux-Falls benchmark [19] shows that the global optimum solutions are found in fewer iterations than those found in some exact algorithms in the literature. (ii) Integration of the objective function among the constraints (as side constraints) provides a commensurate capability to tackle the multi-objective (or multi-criteria) DNDP as well (as the environmental concerns and notions such as sustainability and safety are becoming paramount moving toward multi-objective models is inevitable [2, 20]).

Throughout this article, we assume (i) travel demand is fixed and quantified as a single matrix; (ii) users have a perfect understanding of the travel time, and (iii) neither demand nor travel time change over time. (iv) the decision variables are discrete (binary) not continuous; hence the undertaken problem is a deterministic, static, single-class and discrete network design problem. Appendix A provides a basic discussion on the Outer Approximation method.

## Literature review

A thorough discussion on NDP can be found in [12–14]. A plethora of heuristic methods tailoring to the DNDP has been proposed. Examples are genetic algorithms, ant colony, simulated annealing and particle swarm optimization. Since the proposed methodology lends itself primarily to the class of exact methods, in this section the exact methods developed for the DNDP over the most recent studies are discussed.

As noted above, a general approach to the bilevel programming problems such as the DNDP is to dissolve it to a single-level problem [21–23]. The convention is to replace the lower-level decisions (the TAP) or by its Karush–Kuhn–Tucker (KKT) conditions or to reformulate it as either a variational inequality or complementarity problem [24]. This would result in a single-level MINLP to be solved by methods such as branch-and-bound, Outer Approximation, Lagrangian relaxation, Benders decomposition, descent methods, sequential quadratic programming, penalty function methods, and trust-region methods [25].

Gao, Wu [18] propose the concept of a support function to transform the bilevel DNDP to a single-level MINLP which is then to be solved using Benders decomposition. However [19] have numerically shown that the Benders decomposition may fall into local optimal solutions. Perhaps, one reason could be related to the dual values (or Lagrangian values) of the capacity constraints of the Benders decomposition which are proven not to be unique [26]. Hence, the validity of the Lagrangian based algorithm (The Benders decomposition as well as Lagrangian relaxation methods) needs to be further scrutinized.

Zhang and Gao [27] formulate the TAP in the lower level as a set of complementarity constraints, hence the MNDP and DNDP can be written as a single-level problem. In this study, the optimal size of additional capacity pertaining to the candidate roads is also sought. The solution algorithm is basically a locally convergent algorithm based on the idea of the penalty function, that in turn invokes more investigation when a large-sized network is the case-study.

Similarly, Wang and Lo [28] employ the complementary formulation to arrive at MILP. The solution algorithm is based on a piecewise linear approximation of link travel time functions and it enumerates paths between origin-destination (OD). As the number of ODs increases (which is the case in large-sized networks) number of paths grows exponentially hence the solution algorithm becomes computationally prohibitive. In a similar fashion, Luathep, Sumalee [29] replace the TAP with equivalent variational inequality constraints and employ a linearization scheme as a solution algorithm.

Farvaresh and Sepehri [30] use the KKT conditions of the TAP to arrive at a single-level MILP and then employ some linearization schemes as a solution algorithm. Moreover, they also developed a Branch-and-Bound algorithm based on the seminal work of LeBlanc [31]. In this study, based on the concept of system optimal (SO) a valid (but not necessarily tight) lower bound is calculated aiming to fathom the branch and bound algorithm. Due to the usually significant gap between these bounds [32], the applications are limited to special cases in which such a gap is negligible. Moreover, the branch and bound algorithms are notoriously known to be highly RAM-intensive and computationally expensive methods [33, 34].

Wang, Meng [25] extend the DNDP to also identify the number of additional lanes as additional decision variables. To this end, they adopted a SO-relaxation approach that is assuming drivers not following the shortest path (UE) rather cooperatively minimizing the system’s cost. They develop two solution algorithms based on the organic relation between UE and SO principles. The first method postulates that an SO traffic flow is a closed approximate solution to the UE traffic flow. The second method aims to reduce the gap between the bilevel programming model and the single-level model by adding valid inequalities based on the UE’s Beckmann (objective) function [35].

Fontaine and Minner [4] also adopt a piecewise linearization scheme to transform the bilevel problem to a single-level problem when the TAP is represented with its corresponding KKT conditions. The solution algorithm is developed based on the Benders decomposition and a linearization scheme. In a subsequent work, [36] investigate phases structure of the roads’ maintenance projects as a DNDP which is first linearized and then transformed into a single-level mixed-integer program by using the KKT conditions to be solved with Benders Decomposition. The numerical study shows that this method finds better solutions and faster compared to solving the mixed-integer formulation using a genetic algorithm.

Liu and Wang [1] address the CNDP while accounting for a stochastic user equilibrium traffic flow, that results in a nonlinear nonconvex programming problem which is relaxed via a linearization technique. In contrary to previous methods, a finer piece-wise linearization scheme is adopted, by which a more detailed approximation around global solution is implemented. However, the impact of a number of break points in the proposed scheme on the performance of the algorithm when solving for a large-sized network is a worthy line of research.

Liu and Chen [37] propose an optimization algorithm, the dimension-down iterative algorithm (DDIA), for solving a mixed transportation network design problem (MNDP), The idea of the proposed solution algorithm (DDIA) is to reduce the dimensions of the problem. A group of variables (discrete/continuous) is fixed to optimize another group of variables (continuous/discrete) alternately; then, the problem is transformed into solving a series of CNDPs (continuous network design problems) and DNDPs (discrete network design problems) repeatedly until the problem converges to the optimal solution. For the MNDP with a budget constraint, however, the result depends on the selection of initial values, which leads to different optimal solutions (i.e., different local optimal solutions). The pedagogical dataset of the Sioux falls is used for the numerical tests.

Lu, Atamturktur [38] address the problem of retrofitting at-risk bridges formulated as a DNDP considering stochastic nature of the budget allocation. For numerical analysis, they use the dataset of the Sioux Falls, in which critical factors (such as budget levels) on the retrofit strategy are also analyzed.

González, Dueñas‐Osorio [39] formulate restoration of a partially destroyed system of infrastructure networks as a DNDP subject to budget, resources, and operational constraints for which they develop a MIP model. The authors also propose heuristic methodologies based on a simulation model to identify a reconstruction scenario as well as the order of reconstruction.

Wang and Pardalos [40] develop an active set algorithm to solve the DNDP while assuming that the capacity increase and construction cost of each road is based on the number of lanes. The dataset of the Sioux falls is used for the numerical tests.

As can be seen from the review, the literature has yet to address the DNDP for real-size networks. This study is purposely developed to fill such a gap.

## Mathematical formulations

In this section, the bilevel DNDP is first formulated and discussed. The Outer Approximation algorithm for solving a general single-level MINLP problem is then presented.

### Bilevel network design problem

Definition:

*A*,*A*′: Sets of existing roads and candidate road projects (or shortly called “projects”) respectively.
*N*: set of nodes.
*B*: budget,
*y*_*a*_: binary decisions variable of project *a* ∈ *A*′; 1: to build and 0: no build
*c*_*a*_: construction cost of project *a* ∈ *A*′,
*x*_*a*_,: traffic flow on link *a* ∈ *A*∪*A*′,
*t*_*a*_(*x*_*a*_): travel cost or time or delay of the link *a* ∈ *A*∪*A*′, a non-decreasing differentiable function of link flow *x*_*a*_ (called delay function). We adopt the widely used function developed by U.S. Bureau of Public Roads (BPR): $t_{a}(x_{a})={\bar{t}}_{a}(1+.15{\left(x_{a}/w_{a}\right)}^{4})$ where $t_{a}(0)={\bar{t}}_{a},w_{a}$ are free flow travel time and capacity of link *a* respectively.
$A_{n}^{-},A_{n}^{+}$ set of links starting and ending at node *n* respectively; $A_{n}^{-},A_{n}^{+}\subset A\cup A^{\prime }$
*O*,*D*,Ω: set of origins, destinations, and origin-destination pairs respectively Ω = *O* × *D*.
*q*_*od*_: traffic demand for origin-destination (*o*,*d*) ∈ Ω respectively.
*P*_*od*_: set of paths between origin-destination (*o*,*d*) ∈ Ω.
*h*_*k*_,: total flow and flow on path *k* ∈ *P*_*od*_

The bilevel DNDP may be written as follows (all variables and parameters are considered non-negative unless otherwise stated):

$$\mathrm{Bilevel}\;\mathrm{DNDP}:\;Min\;T(x_{a})=\underset{a\in A\cup A^{\prime }}{\sum }x_{a}.t_{a}(x_{a})$$ (1)

*s.t.*

$$\underset{a\in A^{\prime }}{\sum }c_{a}.y_{a}\leq B$$ (2)

$$y_{a}=0,1\;a\in A^{\prime }$$ (3)

$$Min\;b(x_{a})=\underset{a\in A\cup A^{\prime }}{\sum }\overset{x_{a}}{\underset{0}{\int }}t_{a}(x_{a})dx$$ (4)

$$\underset{k\in P_{od}}{\sum }h_{k}^{}=q_{od},\;\forall (o,d)\in \Omega$$ (5)

$$x_{a}^{}=\underset{i\in \Omega }{\sum }\underset{k\in P_{od}}{\sum }h_{k}^{}\delta_{a,k}^{};\;x_{a}\geq 0\;\delta_{\begin{array}{l} a,k \end{array}}=\{\begin{array}{cc} 1 & a\in k\\ 0 & a\notin k \end{array},\;\forall a\in A\cup A^{\prime }$$ (6)

$$x_{a}\leq U.y_{a}\;a\in A^{\prime }$$ (7)

In the upper level (objective function (1)) the total travel time is minimized. Budget constraint (2) ensures feasibility of the solutions with respect to project construction costs versus available budget. Constraint (3) sets out the binary decision variables. In the lower level, (Eqs (4) to (7)), the UE flow is computed. Eq (7) makes sure that the projects corresponding to no-build decisions (*y*_*a*_ = 0) are excluded from the traffic assignment (*U* is a sufficiently large value and can be considered as ∑_*od*_*q*_*od*_).

### Outer approximation based methodology for the DNDP

A general approach to solve a MINLP is a decomposition technique such as OA. The decomposition-based algorithms run on the assumptions that the respective MINLP problem is essentially a tractable problem (nonlinear programming part) but it has become intractable with the presence of some intractable components (integer variables). Therefore, the problem is decomposed into two parts (nonlinear and mixed-integer) and each is solved separately and alternately while they exchange their results. Therefore, the nonlinear and mixed-integer parts are formulated as primal and dual problems derived from the original MINLP minimization problem: (i) the primal problem is constituted as solving the original problem with a feasible solution for the binary variables; hence it renders an upper bound to the original problem. (ii) given the solution results of the primal problem, the dual problem is designed as a mixed-integer linear programming (MILP) to render a new solution for the binary variables. Since it is a dual problem it also gives a lower bound to the original problem. This process carries on until the gap between these upper bound and lower bounds are sufficiently small (termination condition). It has numerically been observed that a significant effort is made to reduce the gap while the optimum solution is found earlier [18]. In other words, the optimum solution is found in the intermediate iterations while the following iterations are carried out to prove that no better solution is found.

Now we turn our attention to the fact that the undertaken DNDP is NP-hard. This means that finding the optimum solution for the large-size networks is almost impossible. Hence, why we are trying to prove whether an optimum solution is found or not? So, it is highly justified to spare the solution methods from additional exhaustive iterations to get the afore-mentioned gap to vanish. Therefore, we exploit the pre-known fact that the problem is NP-hard and turn the complexity of the problem to our advantage. As the result, in this study, exempting the solution algorithm from narrowing down the gap is the key leverage in addressing the DNDP for large-size networks. The afore-mentioned gap has two bounds, upper and lower: the effort to solve the primal problem (upper bound) remains intake since it renders a feasible solution; instead the relaxation is exerted in the dual problem (lower bound). Because of ignoring the gap between the upper bound and lower bound as a termination criterion, the proposed methodology can no longer be classified as an exact methodology (as mentioned before, the best description for it is a hybrid exact-heuristic method).

### Single-level programming for DNDP

To establish the methodology, we first proceed to develop a single-level problem. The UE formulation in the lower level is considered as the base and the objective function along with the remaining binary-related-constraints of the upper level (Eqs (1) to (3)) are integrated into as additional constraints:

DNDP-UE-MINLP:

$$MinT(x_{a})=\underset{a\in A\cup A^{\prime }}{\sum }\overset{x_{a}}{\underset{0}{\int }}t_{a}(x_{a})dx$$ (8)

$$S.t.\;\underset{k\in P_{od}}{\sum }h_{k}^{}=q_{od},\;\forall (o,d)\in \Omega$$ (9)

$$x_{a}^{}=\underset{i\in \Omega }{\sum }\underset{k\in P_{od}}{\sum }h_{k}^{}\delta_{a,p}^{}\;\delta_{a,p}=\{\begin{array}{cc} 1 & a\in p\\ 0 & a\notin p \end{array},\;\forall a\in A\cup A^{\prime }$$ (10)

$$x_{a}\leq U.y_{a}$$ (11)

$$y_{a}=1\;or\;0,\;a\in A^{\prime }$$ (12)

$$\underset{a\in A^{\prime }}{\sum }c_{a}.y_{a}\leq B$$ (13)

$$\underset{a\in A\cup A^{\prime }}{\sum }x_{a}.t_{a}(x_{a})\leq ub_{*}^{i}$$ (14)

where Eqs (8) to (13) have already been introduced. In Eq (14), $ub_{*}^{i}$ is the least total travel time (value of the objective function (1)) found so far at iteration *i* (in other words, it is the best upper bound value found so far, better known as “incumbent value”). It is worth noting that, it is an iterative process, and we can easily keep track of the best solution found (best upper bound value) throughout. The algorithm will start with an educated guess for a feasible binary solution or at least with a do-nothing scenario (*y*_*a*_ = 0,*a* ∈ *A*′: no project is constructed) to solve the corresponding UE-TAP. Hence the best total travel time is constantly computed and saved as $ub_{*}^{i}$. Therefore, constraint (14) forces the algorithm to try to reach a better solution in each iteration (i.e. the better solution is the one with lower upper bound). The above DNDP-UE-MINLP has an important property: both the Beckmann objective function and the constraints are convex [41] and differentiable. Therefore, the OA is a good solution algorithm to the MINLP problem [42, 43].

It is important to note that linearization component of the OA algorithm is devised to transform an (almost) unsolvable mixed-integer “non-linear” problem to a solvable mixed-integer “linear” problem. This is the main thrust of decomposition methods such as Lagrangian relaxation, Benders decompositions and OA [43]. In the end, the only useful outcome of the OA is a new binary feasible solution. These new binary feasible solutions are then used to solve a fully-fledged traffic assignment with no linearization scheme. In other words according to the seminal article [44] the linearization does not compromise arriving at a globally optimal solution. We now proceed to establish the OA solution algorithm for the DNDP-MINLP.

### Outer approximation for DNDP-UE-MINLP

In this section, we explain how to linearize each element of the DNDP-UE-MINLP. Consider *Y*^*i*^ ∈ *V* is a feasible solution for the binary variables at iteration *i*. Given *Y*^*i*^ ∈ *V* solving the UE-TAP is equivalent to solving the *NLP*(*y*^*i*^) of the DNDP which gives $x_{a}^{i},a\in A\cup A^{\prime }$ links flow solution at iteration *i*. Now, given $x_{a}^{i}$, the master outer approximation problem can be established. We first start the linearization with the objective function (8) denoted by *z* which can be written as:

$$z\geq \underset{b^{i}}{\underset{︸}{\underset{a\in A\cup A^{\prime }}{\sum }\overset{x_{a}^{i}}{\underset{0}{\int }}t_{a}(x_{a})dx}}+\underset{a\in A\cup A^{\prime }}{\sum }\underset{t_{a}(x_{a}^{i})}{\underset{︸}{d(\overset{x_{a}^{i}}{\underset{0}{\int }}t_{a}(x_{a})dx)/dx}}.(x_{a}-x_{a}^{i})$$ (15)

where the first term in the right-hand side is the value of the Beckmann function denoted by *b*^*i*^ and the derivative of the integral in the second term is equal to the argument under the integral which is the travel time of the respective link $t_{a}(x_{a}^{i})$. Hence, by splitting the sigma and bring all the variables (*x*_*a*_,*z*) on one side, the cut related to the objective function are:

$$\underset{a\in A\cup A^{\prime }}{\sum }t_{a}(x_{a}^{i}).x_{a}-z\leq \underset{ub^{i}}{\underset{︸}{\underset{a\in A\cup A^{\prime }}{\sum }t_{a}(x_{a}^{i}).x_{a}^{i}}}-b^{i}$$ (16)

where the first term on the right-hand side is *ub*^*i*^ the total travel time/cost spent on the network which is the value of the objective function of the original problem (Eq (1)). Hence it is an upper bound found at iteration *i*. At each iteration (*i*) after solving the UE-TAP, the total travel time of the current scenario is saved as *ub*^*i*^ up to this iteration the least travel time is also updated and represented by $ub_{*}^{i}$.

Constraints (9) and (10) ensure that the resultant flow meets the travel demand over paths connecting all origins to all destinations. In network flow terminology, flow pertaining to each origin-destination pair is called a commodity, hence, constraints (9) and (10) constitute a multi-commodity traffic flow pattern. Enforcing multi-commodity flows by link-based linear constraints requires a high number of constraints and variables. In fact, for each OD (or commodity) one has to establish the conservative flow constraints which result in |*N*| constraints and |*A*∪*A*′| variables (note that, for the multi-commodity formulation, number of constraints and variables will be |Ω|.|*N*| + |*A*∪*A*′| and (|Ω|+1).|*A*∪*A*′| respectively, Ω is set of OD pairs). As such, the size of the resulting MILP problem increases drastically which makes it intractable. The outcome of the afore-mentioned MILP is a new solution for the binary variables *y*^*i*^ and the traffic flow $x_{a}^{i}$. Based on the *y*^*i*^, its corresponding UE-TAP is solved and again a new solution for the traffic flow is obtained. Therefore, the $x_{a}^{i}$ out of the MILP is redundant. Consequently, in the formulation of the MILP, we spare less effort for the $x_{a}^{i}$, that is, not all constraints of the multi-commodity flow are included in the MILP. As such, we relax the multi-commodity formulation to a single-commodity formulation by only holding conservative flow at nodes. Thus, we replace constraints (9) and (10) with equivalent link-based conservative flow constraint:

$$\underset{a\in A_{n}^{+}}{\sum }x_{a}-\underset{a\in A_{n}^{-}}{\sum }x_{a}=\{\begin{array}{cc} -\sum_{o\in O}q_{on} & \;n\in D\\ +\sum_{d\in D}q_{nd} & \;n\in O\\ 0 & \;o.w. \end{array}\;n\in N$$ (17)

According to constraint (17), all origins and destinations are aggregated to a single (dummy) origin and a single (dummy) destination. Again, we relax the multi-commodity (or path-base) formulation (9) and (10) to a single-commodity (or link-base) (17). It is important to note that the traffic flows of the multi-commodity will be different than that of the single-commodity, but, it does not compromise our methodology since a fresh and complete traffic flow solution will be found later (see Step 1 of the proposed algorithm at the end of this section).

Constraints (11), (12) and (13) as well as (17) are carried over intact to the master outer approximation problem since they are already linearized. According to Taylor’s first order series, a linear approximation of the last constraint (14) can be written as:

$$0\geq \underset{ub^{i}}{\underset{︸}{\underset{a\in A\cup A^{\prime }}{\sum }x_{a}^{i}.t_{a}(x_{a}^{i})}}-ub_{*}^{i}+\underset{a\in A\cup A'}{\sum }\underset{\begin{array}{l} x_{a}^{i}.t_{a}^{'}(x_{a}^{i})+t_{a}(x_{a}^{i})\\ or\;if\;t_{a}\;complies\;BPR:\\ 5t_{a}(x_{a}^{i})-4t_{a}(0) \end{array}}{\underset{︸}{\{d(\underset{a\in A\cup A^{\prime }}{\sum }x_{a}.t_{a}(x_{a})-ub_{*}^{i})/dx}}.(x_{a}-x_{a}^{i})\}$$ (18)

As mentioned before, the first term is *ub*^*i*^ the total travel time. It is easy to show that the derivative term results in system optimal travel time [41], that is: $x_{a}^{i}.t_{a}^{'}(x_{a}^{i})+t_{a}(x_{a}^{i})$. Alternatively, for the favorite BPR delay functions, the system optimal travel time can be easily calculated as $5t_{a}(x_{a}^{i})-4t_{a}(0)$ which is used henceforward without loss of generality. By reordering the variables on the left side and the constant values on the right side of the inequality we get:

$$\underset{a\in A\cup A'}{\sum }(5t_{a}(x_{a}^{i})-4t_{a}(0))x_{a}^{}\leq ub_{*}^{i}-ub^{i}+\underset{a\in A\cup A'}{\sum }(5t_{a}(x_{a}^{i})-4t_{a}(0))x_{a}^{i}$$ (19)

Now, the above-linearized constraints can be put together and the master outer approximation of the user equilibrium traffic assignment problem is configured:

$$DNDP-UE-MOA^{i}:\min\;z$$

$$s.t.\;\underset{a\in A\cup A^{\prime }}{\sum }t_{a}(x_{a}^{k}).x_{a}-z\leq ub^{k}-b^{k}\;k\in T^{i}$$ (20)

$$\underset{a\in A\cup A'}{\sum }(5t_{a}(x_{a}^{k})-4t_{a}(0))x_{a}^{}\leq ub_{*}^{k}-ub^{k}+\underset{a\in A\cup A'}{\sum }(5t_{a}(x_{a}^{k})-4t_{a}(0))x_{a}^{k}\;k\in T^{i}$$ (21)

$$x_{a}^{}\leq U.y_{a}^{}$$ (22)

$$y_{a}^{}=1\;or\;0,\;a\in A^{\prime }$$ (23)

$$\underset{a\in A^{\prime }}{\sum }c_{a}.y_{a}^{}\leq B$$ (24)

$$\underset{a\in A_{n}^{+}}{\sum }x_{a}-\underset{a\in A_{n}^{-}}{\sum }x_{a}=\{\begin{array}{cc} -\sum_{o\in O}q_{on} & \;n\in D\\ +\sum_{d\in D}q_{nd} & \;n\in O\\ 0 & \;o.w. \end{array}\;n\in N$$ (25)

$$\underset{a\in Y1^{k}}{\sum }y_{a}-\underset{a\in Y0^{k}}{\sum }y_{a}\leq |Y1^{k}|-1,\;Y1^{k}=\{a|y_{a}^{k}=1\};\;Y0^{k}=\{a|y_{a}^{k}=0\};k\in T^{i}$$ (26)

Given $x_{a}^{i},a\in A\cup A^{\prime }$ the DNDP-UE-MOA^i^ renders *z*, *x*_*a*_,*a* ∈ *A*∪*A*′ and set of binary solutions *y*_*a*_,*a* ∈ *A*∪*A*′. The last constraint (26) proposed by [45] ensures that in each iteration a new set of binary solutions is obtained. The above-relaxed problem renders a lower bound (*z*) to the Beckmann value of the UE-TAP (*b*^*k*^) derived from solving the DNDP with a feasible binary solution. The algorithm normally terminates once no new binary solution is found or the lower bound reaches the upper bound. For large-sized networks, the former is unlikely to occur, nor does the latter since it is reformulated to a linear approximation and single-commodity constraints. Therefore, the termination condition is set as a user-specified maximum number of iterations. The algorithm is summarized as follows:

**Step 0.** Initialization: Set maximum iteration *i*_max_, current iteration *i* = 1 and choose a feasible solution for decision variables *y*^*i*^ ∈ *Y* (for instance *y*^*i*^ = (0,0..0) the do-nothing or no-build scenario). Initialize upper bound as $ub_{{}^{*}}^{0}=-\infty$, (as mentioned before, no need to keep records of the lower bound).
**Step 1**. Calculate Upper bound: Given *y*^*i*^, solve UE-TAP to obtain *x*^*i*^ and the Beckmann value *b*^*i*^ followed by computing the total travel time $ub_{}^{i}=\sum_{a}t_{a}(x_{a}^{i}).x_{a}^{i}$. Update the incumbent value of the best solution found so far by $ub_{*}^{i}=\min\{ub_{*}^{i-1},ub_{}^{i}\}$. Save the binary solutions *y*^*i*^ as best solution denoted by y* if it was found the best solution so far.
**Step 2.** Calculate Lower bound and obtain a new binary solution: Given *x*^*i*^ solve the master problem DNDP-UE-MOA^i^ to obtain: *z*^*i*^,*x*^*i*+1^,*y*^*i*+1^.
**Step 3.** Termination: if *i* > *i*_max_ stop and y* is the optimal solution, otherwise set *i* ≔ *i* + 1 and go to Step 1.

**Fig 1:**
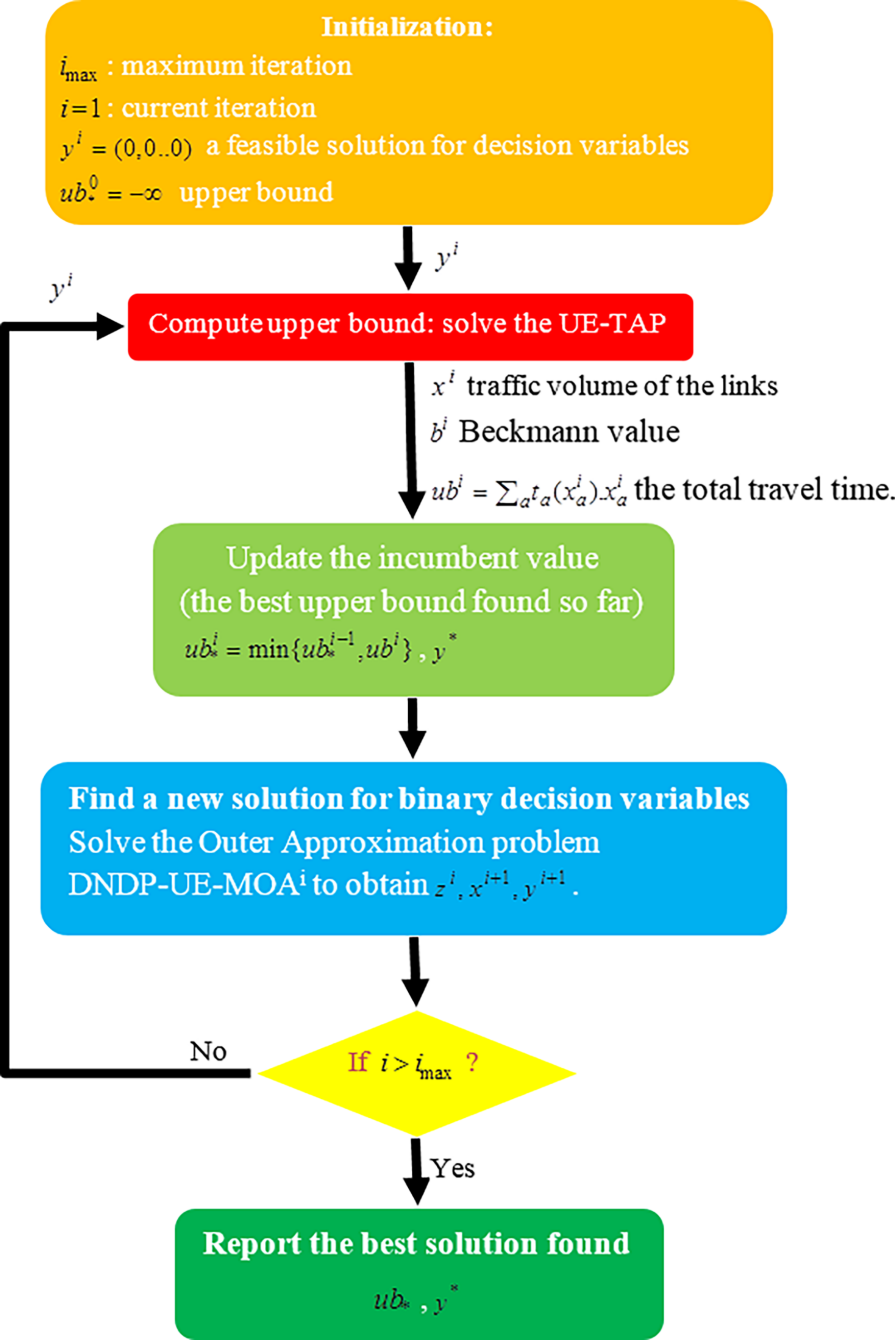
A snapshot of the methodology.

**Fig 1** provides an overview of the methodology. The detail of the proposed algorithm is further discussed using the following example.

#### Example 1 (a single OD network)

**Fig 2:**
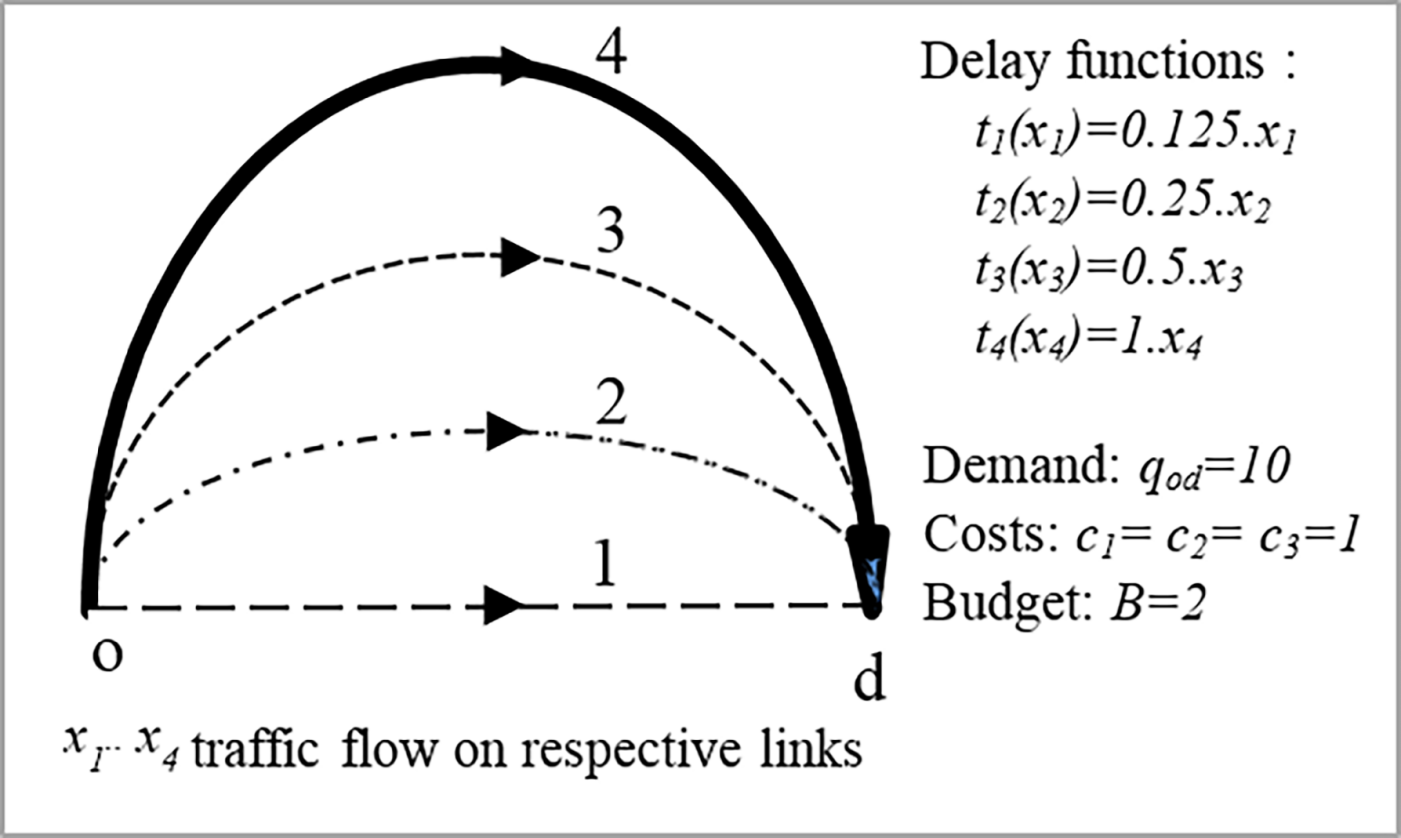
Example 1, a single OD network.

Consider a network as shown in **Fig 2** consists of a single road (#4) connecting an origin-destination pair with the travel demand of *q*_*od*_ = 10. The plan is to ease traffic by constructing the maximum of two additional roads from three candidate road projects number 1 to 3. The associated construction costs and available budget are *c*_1_ = *c*_2_ = *c*_3_ = 1; *B* = 2. The roads delay functions are presented in **Fig 2**. The question is: which subset of the candidate roads is the best choice. Set *i*_max_ = +∞ to capture all the possible solutions. The algorithm starts with do-nothing scenarios Y^0^ = (0,0,0) based on which the UE-TAP is solved and it results in traffic flow: X^0^ = (0,0,0,10), Beckman value:50 and total travel time:100. The first DNDP-UE-MOA problem is tabulated from row number 1 to 8 in Table 1. Constraints of rows 1 to 4 correspond to discrete constraints, and constraint of row 5 represents the only conservative constraint consistently shown by inequality sign: −*x*_1_−*x*_2_−*x*_3_−*x*_4_ ≤ −10 (since it is a minimization problem the afore-mentioned inequality is always binding). Constraints of rows 6,7 and 8 are the first cuts corresponding to the constraints (20), (21) and (26) of the general formulation respectively.

**Table 1:** Example 1; original Outer Approximation (OA) algorithm and the iterative results.

no	iteration no	DNDP-UE-Outer Approximation tableau									DNDP-UEMOA Results			Results of Solving UE-TAP (Traffic Assignment)			
		x1	x2	x3	x4	y1	y2	y3	z	RHS*	z^i^	X^i^	Y^i^	X^i^	Beckmann Value	Total Travel Time	Incumbent Value
1	0	1				-10											
2			1				-10										
3				1				-10									
4						1	1	1		2							
5		-1	-1	-1	-1					-10							
6	1				10				-1	50	0	0,10,0,0,0	0,1,0	0,8,0,2	10	20	20
7					20					200							
8						-1	-1	-1		-1							
9	2		2		2				-1	10	0	10,0,0,0	1,0,0	8.9,0,0,1.1	5.6	11.1	11.1
10			4		4					40							
11						-1	1	-1		0							
12	3	1.1			1.1				-1	5.6	0	0,0,10,0	0,0,1	0,0,6.7,3.3	16.7	33.3	11.1
13		2.2			2.2					22.2							
14						1	-1	-1		0							
15	4			3.3	3.3				-1	16.7	0	5,5,0,0	1,1,0	6.1,3.1,.8	3.8	7.7	7.7
16				6.7	6.7					44.4							
17						-1	-1	1		0							
18	5	0.7	0.7		0.7				-1	3.8	0	5,0,5,0	1,0,1	7.3,0,1.8,.9	4.5	9.1	7.7
19		1.5	1.5		1.5					15.4							
20						1	1	-1		1							
21	6	0.9		0.9	0.9				-1	4.5	0	0,5,5,0	0,1,1	0,5.7,2.8,1.4	7.1	14.3	7.7
22		1.8		1.8	1.8					16.8							
23						1	-1	1		1							

* RHS means right-hand side of the inequality constraints with inequality sign of "≤"

The result of solving the DNDP-UE-MOA is also shown in the table where the flow and the binary solutions are found X^1^ = (0,10,0,0), Y^1^ = (0,1,0) respectively. The resultant binary solution is used for solving the UE-TAP to obtain accurate traffic flow and supersedes the previously found traffic flow which was based on linear approximation: X^1^ = (0,8,0,2). Furthermore, the Beckmann value, the total travel time and the updated incumbent value are derived as 10,20 and 20 respectively. The calculation carries on until no new binary solution is found. As shown in Table 1, the optimum solution is found at iteration 4: Y* = Y^4^ = (1,1,0).

### Refined OA algorithm

With regard to the natural course of the problem and the sequential process of the algorithm shown for the above example there are two important observations:

I In the first three iterations, the algorithm reached the solutions with only one project selected while the budget is for two projects. Provided the candidate projects have been selected wisely, a good solution is expected to consume the budget to its full. Hence the more projects contribute to the solution the better the solution becomes.
II The algorithm started with a null solution (do-nothing scenarios Y^0^ = (0,0,0)). Is there any better-educated guess to start with? If so would it be better to start with that educated guess?

The proposed algorithm is rectified to accommodate these two conceivable points as follows: (i) in order to improve the algorithm to assign value 1 to more binary variables (*y*_*a*_), the objective function of the DNDP-UE-MOA is changed to: $\min z-\sum_{a\in A^{\prime }}y_{a}$. (ii) for the starting binary solution, one can obtain a good solution as follows: establish a network scenario including all the projects and irrespective of their budget and costs. Solve the UE-TAP and obtain *x*_*a*_,*a* ∈ *A*′ the traffic volume for each of the projects. Projects with higher traffic volume have a higher likelihood to be selected in the optimum solution. Normalize the traffic volume to the capacity and associated cost to obtain a fair merit index. Hence the merit index is defined as *x*_*a*_/*c*_*a*_/*w*_*a*_,*a* ∈ *A*′. Therefore, first, based on the merit index, sort the candidate projects in descending order. Second, select the projects from the top of the sorted list until the budget is depleted; this renders an initial solution which is also called “intuitive solution”.

### Convergence proof

Duran and Grossmann [44] set out three conditions to ensure convergence of the OA algorithm as follows: C1: solution space on continuous variables (i.e. link flow *x*_*a*_) must constitute a nonempty, compact and convex set. C2: A constraint qualification must hold on continuous variables for any feasible solutions of integer variables. C3: Functions (objective function and constraints) associated with continuous variables must be convex and once continuously differentiable.

Let us rewrite the DNDP-UE-MINLP problem, give a feasible binary solution as:

$$MinT(x_{a})=\underset{a\in A\cup A^{\prime }}{\sum }\overset{x_{a}}{\underset{0}{\int }}t_{a}(x_{a})dx$$ (8, repeated)

$$S.t.\;\underset{k\in P_{od}}{\sum }h_{k}^{}=q_{od},\;\forall (o,d)\in \Omega$$ (9, repeated)

$$x_{a}^{}=\underset{i\in \Omega }{\sum }\underset{k\in P_{od}}{\sum }h_{k}^{}\delta_{a,p}^{}\;\delta_{a,p}=\{\begin{array}{cc} 1 & a\in p\\ 0 & a\notin p \end{array},\;\forall a\in A\cup A^{\prime }$$ (10, repeated)

$$\underset{a\in A\cup A^{\prime }}{\sum }x_{a}.t_{a}(x_{a})\leq ub_{*}^{i}$$ (14, repeated)

Since we solve basically a UE-TAP (i.e. Eqs 8, 9 and 10) after finding a feasible binary solution, condition C1 is subsequently upheld [35, 41, 46]. As for C2, it is sufficient to prove that the solution domain of the above problem ((i.e. Eqs 8, 9, 10 and 14)) is convex. In other words, the constraint qualification is always satisfied for a convex feasible region where at least a feasible solution exists [47]. Note: the do-nothing scenario is always a feasible solution. To do so, we just need to prove that constraint (14) represents a convex set, since constraints (9, 10) shape up solution space of the UE-TAP and it is already proven to be convex [35] (note that, intersection of two convex sets itself is a convex set [48]). As for C3, all functions (pertaining to UE-TAP) are proven to be convex, and what is left is the constraint (14). Consequently, the whole proof effort boils down to proving that constraint (14) constitutes a convex set which is discussed as follows. There are theorems on convexity that are used in our proof [48–51]:

a a constraint of the form "convex function of x should be less than or equal to a constant w" defines a convex set
b a sum of convex functions is a convex function.
c a function of one variable is convex if its derivative is an increasing function.
d a function is increasing if its derivative is non-negative.
e for a non-decreasing function, the second-order derivative is non-negative if and only if the function is convex.

In our case, we have additive terms on the left-hand side of the form *x*_*a*_.*t*_*a*_(*x*_*a*_), where *t*_*a*_(*x*_*a*_) is the travel time function, and *x*_*a*_ is the flow on the link *a* ∈ *A*∪*A*′. If we prove each additive term is convex, then, according to theorem (a, b) the bounded sum function (i.e. $\sum_{a\in A\cup A'}x_{a}.t_{a}(x_{a})$) delineates a convex set and hence end of the proof. To do so the derivative of an additive term with respect to *x*_*a*_ is $t_{a}(x_{a})+x_{a}.t_{a}^{\prime }(x_{a})$ where $t_{a}^{\prime }$ is the derivative of *t*_*a*_. According to theorem (c) If we show that this is an increasing (in fact, non-decreasing) function of *x*_*a*_ then, it means that *x*_*a*_.*t*_*a*_(*x*_*a*_) is a convex function. Hence we just need to show that the second term (i.e. $x_{a}.t_{a}^{\prime }(x_{a})$) is increasing in *x*_*a*_ since the first term is already assumed to be so. According to theorem (d), we show that its derivative (i.e. $t_{a}^{\prime }(x_{a})+x_{a}.t_{a}^{\prime \prime }(x_{a})$) is non-negative. It is evident that the first term is non-negative since travel time function is assumed convex and hence its derivative is already non-negative. For the second term, we just need to show that $t_{a}^{\prime \prime }(x_{a})\geq 0$ since *x*_*a*_ ≥ 0 which is proved based on theorem (e).

To summarize: if *t*_*a*_ is a non-decreasing, differentiable, and convex function then a constraint of the form of $\sum_{a\in A\cup A'}x_{a}.t_{a}(x_{a})\leq ub_{*}^{i}$ will define a convex set—moreover a closed one (the boundary is included) because the function is continuous.

## Numerical evaluations

In this section, we first apply both the original and refined OA algorithms to Gao’s 12-nodes network [18] and Sioux Falls network to compare them with their peers in the literature. We finally apply the algorithm to the large-size network of the city of Winnipeg, Canada.

### Example 2: Gao’s network

**Fig 3:**
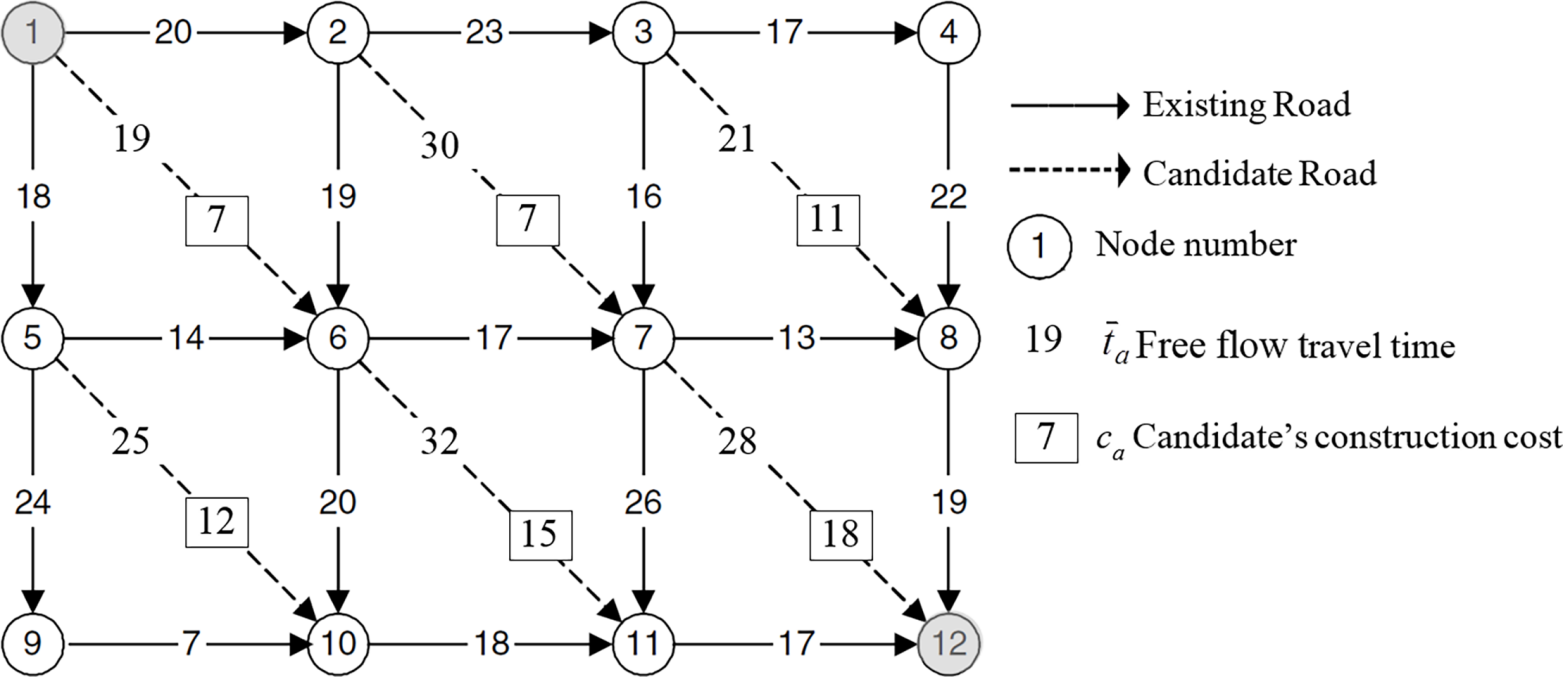
Gao’s test network.

**Fig 3** illustrates the example network developed by Gao, Wu [18] with one OD pair (1,12) and the travel demand of *q*_1,12_ = 20. The delay function is $t_{a}={\bar{t}}_{a}+.008x_{a}{}^{4}$. This can be rearranged as per BPR format: $t_{a}={\bar{t}}_{a}(1+.15{\left(x_{a}/w_{a}\right)}^{4})$ where the capacity of the link is. There are 6 candidate projects with the total cost of 70. Gao, Wu [18] developed and applied General Benders Decomposition (GBD) to various budget levels and the results are summarized in Table 2. As discussed in the literature review section, at each iteration of the GBD, two problems: the UE problem and a MILP are solved. Hence, it is analogous to the OA proposed in this study. As such, a number of iterations to reach the optimum solution is considered for evaluation.

**Table 2:** Example 2; Gao’s network: Results of GBD [18] and original/refined OA.

Budget*	Optimal** solution	Number of feasible solutions	Incumbent Value	GBD method: Optimum solution was found at iteration***	GBD-BB method: Optimum solution was found at iteration	Proposed; OA method:	
						Original OA: Optimum solution was found at iteration	Refined OA: Optimum solution was found at iteration
10	100000	3	4076	2	3-2-0****	1	0*****
20	101000	12	3952	4	3-5-0	3	0
30	100001	26	2668	6	4-3-2	1	2
40	100101	41	2524	4	4-5-2	4	3
50	101101	52	2404	4	4-6-5	3	2
60	101111	61	2281	5	4-5-2	4	3
70	111111	64	2256	5	3-1-0	1	0

* Total construction costs is 70

** the digits of the binary strings represents the following two-ways candidates respectively: (1,6), (5,10), (2,7), (6,11), (3,8),(7,12)

*** Gao and Wu [18]

**** x-y-z: x: no. of UE solved, y: no. of Benders (lower bound) solved, z: Benders iteration at which optimum solution was found

***** iteration zero refers to the intuitive (or initial) solution, the sorted projects as per the merit index is: (1,6),(2,7),(7,12),(5,10),(3,8),(6,11)

Table 2 also presents the iteration at which the optimum solutions were found over various budget level2 for the GDB as well the two types of the OA developed in this study: original and refined OA. As is evident, the OA reaches the optimum solution sooner than the GBD. Moreover, the refined OA (OA with the intuitive solution and refined objective function) has significant superiority over the original OA algorithm.

#### Example 3, Sioux-Falls network

The proposed algorithm is applied to the Sioux-Falls first introduced by [31] and recently used by Farvaresh and Sepehri [19] employing a Branch-and-Bound (BB) method. There are 5 two-ways candidate roads with a total cost of 4325. At each iteration of the BB, two problems are solved: (i) a UE-TAP and (ii) an Outer Approximation problem to solve a MILP for SO network design problem. Hence, it is similar to the OA proposed in this study. As such, a number of iteration to reach the optimum solution is considered for evaluation. Similarly, Table 3 presents the iteration at which the optimum solutions were found over various budget levels for both methods. As it is evident from Table 3, the refined OA by far surpasses the original OA. This time, the refined OA lags the BB in one out of three accounts (B = 3000). Nonetheless, there is an important observation worth noting as discussed in the following exposition.

**Table 3:** Example 3; Sioux-Falls: Results of BB [19] as well as original and refined OA.

Budget*	Optimal solution**	Number of feasible solutions	Incumbent Value	GBD-BB method: Optimum solution found at iteration***	B-B method: Optimum solution found at iteration****	Method proposed in this study; OA method:	
						original OA: Optimum solution found at iteration	Refined OA: Optimum solution found at iteration*****
2000	00101	14	158.4158	4-7-1	27	5	1
3000	00111	23	113.2047	4-9-1	39	20	8
4000	10111	32	94.1993	4-7-2	65	22	1

* Total construction costs is 4325

** The digits of the binary strings represents the following two-ways candidates respectively: (6,8), (7,8), (9,10), (10,16), (13,24)

*** Bagloee, Sarvi and Patriksson [53]

****Farvaresh and Sepehri [19]

**** the sorted list of the candidate projects as per the merit index for the intuitive solution is (9,10),(6,8),(13,24),(7,8),(10,16)

Such an efficient result from a BB algorithm reported by Farvaresh and Sepehri [19] (BB-FS in short) contradicts the literature [14, 25, 31]. It is important to note that the key success in BB algorithms is the cuts made to the solution space because of the times at which the lower bounds are found above the incumbent values. The lower bound in the BB-FS is the total travel time of a System Optimal traffic flow sought by solving a SO network design problem, while the incumbent value is the total travel time of UE traffic flow. Usually, the gap between these two bounds is very large [32, 52], hence it is highly unlikely to achieve any cut to the solution space. Therefore, we computed the total travel time of both SO and UE traffic flow for the undertaken Sioux-Falls network which were found almost equal. These are 785.284 and 786.178 for SO and UE respectively with the relative gap was 0.0001. This shows that the Sioux-Falls undertaken by Farvaresh and Sepehri [19] is biased to a very rare situation in which both SO and UE traffic flows are identical. In other words, the BB-FS for the Sioux-falls is relaxed to solving the SO network design problem; hence its efficiency has yet to be investigated.

#### Example 4: Winnipeg large-scale network

**Fig 4:**
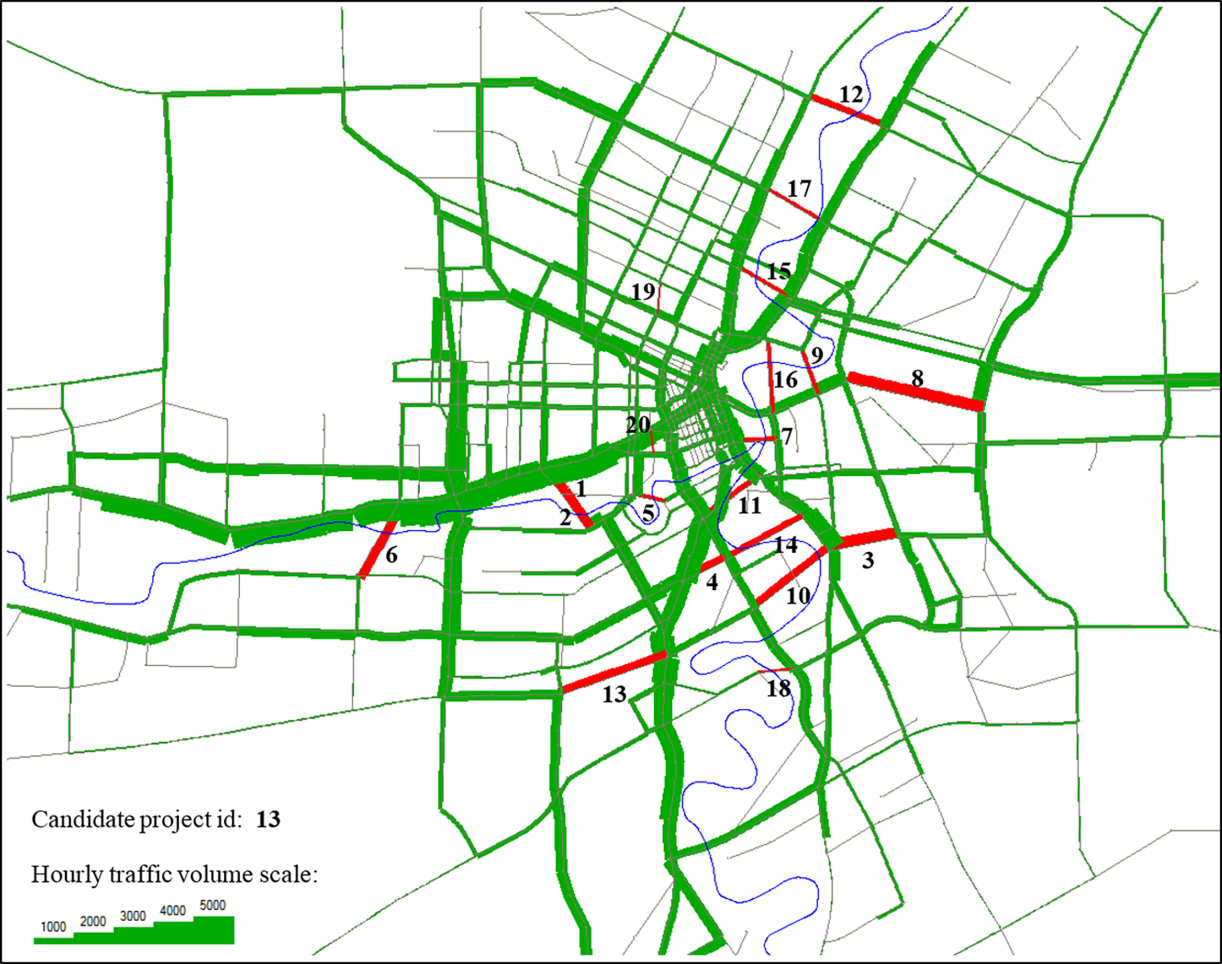
Winnipeg transport network with 20 candidate road projects and hourly traffic volume.

Real-size transportation data of the city of Winnipeg, Canada widely used in the literature [54] is utilized for the numerical tests in this work (it is also provided in EMME 3 [55] transport planning software). The case study comprises of 154 zones, 903 nodes, and 2528 directional links. Total hourly passenger car equivalent demands are 56,219. This dataset is made available to the research community to be used as a benchmark (see this link [56]). As for the computational technology, we employ a desktop computer with Intel(R) Xeon(R) 3.70 GHz and 64.0 GB RAM. The algorithm is coded with Visual Basic linked to MS-Excel as an interface (MS-Access to save/retrieve computation data) and EMME 3 to solve the traffic assignment. the code is linked and synchronized to MATLAB 14a [57] to solve the MILP problems using newly released module “intlinprog”. The delay functions associated with the links conform to the BPR type. We consider 20 road projects with free flow travel time and capacity of 0.456 min and 1700 vph respectively. Table 4 presents the candidate projects sorted based on their merit index in descending order. **Fig 4** shows the locations of the candidate projects and the extent of the undertaken case study on which UE traffic volumes are also depicted. These projects are wisely set forth to complement the ring roads around the central business district and over the river.

**Table 4:** Winnipeg case study, candidate road projects sorted based on the merit index.

Id	1	2	3	4	5	6	7	8	9	10	11	12	13	14	15	16	17	18	19	20
I-node	595	513	325	424	420	551	301	288	297	330	304	177	441	327	168	299	173	335	739	889
J-node	602	595	330	437	592	610	1035	294	1057	428	423	853	494	424	784	1058	829	449	774	898
Cost*	0.59	0.79	1.3	0.86	0.58	1.51	0.75	2.5	0.88	1.73	1.29	1.52	2.04	1.61	1.09	1.35	1.24	0.64	0.6	0.42
Traffic volume	1648	1648	2010	1256	661	1610	685	2011	668	1303	949	1023	1367	983	645	654	553	285	240	0
Merit Index**	2793	2086	1546	1461	1139	1066	913	804	759	753	736	673	670	611	591	484	446	446	400	0

* Total Cost: 23.29

** Provided that the capacity of the projects are same (1700 vph) the merit index is simply calculated as traffic volume/Cost

The lengths of roads are considered as corresponding construction costs which mount to total costs of a C = 23.29 unit of money. With respect to the total cost (C), we take 10 levels of the budget (B) into the analysis as follows: B/C = 10%, 20%..100%. We first carried out an exhaustive enumeration to find the global optimum solution for each budget level. The enumeration consists of 2^20 = 1,048,576 solving traffic assignment problems with a relative gap of 1% which lasted 36 days. The refined OA algorithm is run only for 100 iterations and the results for various budget levels are shown in Table 5.

**Table 5:** Winnipeg case study: Results of refined OA pertaining to up to only 100 iterations.

B/C %	Budget	Number of feasible solutions	Optimal Solution				Application results of the Refined OA		
			Optimal solution string	Cost	Budget used (%)	Incumbent Value	Iteration (< 100) at which optimal solution was found	CPU time (min)	Gap distance of the best solution found to the optimal solution (%)
10	2.329	225	00001000000100000000	2.1	90	1238414	5	13.92	0
20	4.658	6381	00001001000100000000	4.6	99	1232135	53	14.93	0
30	6.987	54879	11000001100100000100	6.9	99	1226368	91	14.90	0
40	9.316	222664	11001011000100110000	9.2	98	1223845	Not found	14.55	0.01593
50	11.65	524288	11001111000110010000	11.6	100	1220833	Not found	13.23	0.11844
60	13.97	825912	11001111101110001000	13.7	98	1218753	Not found.	13.82	0.17887
70	16.3	993697	11111101110110100100	16.0	98	1216904	Not found.	13.28	0.17988
80	18.63	1042195	11111111110110011100	18.3	98	1214734	Not found.	15.00	0.16152
90	20.96	1048351	11111111110111011110	20.5	98	1214006	47	15.05	0
100	23.29	1048576	11111111110111111101	21.4	92	1213749	13	12.37	0

**Fig 5:**
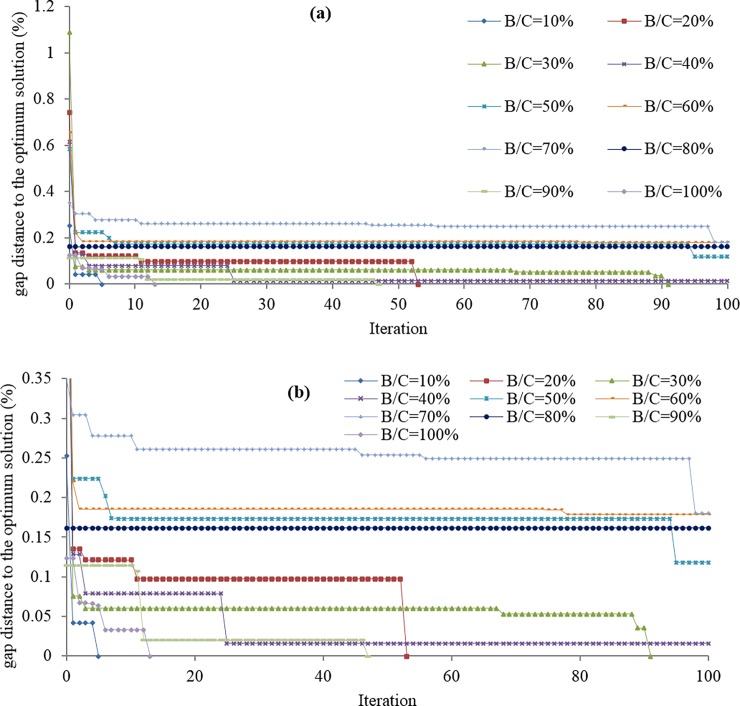
Winnipeg Case Study, Variation of the results for different budget levels over 100 iterations: (a) an overall view, (b) a closer view.

As seen in Table 5, the proposed algorithm was able to find the optimal solutions for half of the budget levels within the 100 iterations. The gap distances of the solutions found to the respective optimal solutions (in percentage) are also shown in **Fig 5**. As discussed earlier the algorithm starts with the intuitive solution. Hence, the first points slated on the y-axis of the graphs represent the intuitive solutions for the respective budget levels. Table 5 also reports on the CPU times which are found in 14 minutes. The following remarks can be inferred from the results: (i) the significant distances between the first points (intuitive solutions) and the following points for almost all the budget levels show the efficacy of the proposed algorithm, (ii) in the earlier iterations a significant lump of the gap is filled, (iii) in case the optimal solution is not found within 100 iterations, the algorithm renders a very close solution to the optimal solution which is also shown in the last column of Table 5.

### A comparison to an exact method

To shed more lights on the computational efficacy of the proposed algorithm, Table 6 presents numerical results pertaining to Benders decomposition algorithm hybridized with a RAM-efficient Branch and bound algorithm (BD-BB) [53] a globally optimal algorithm, which is applied to the same case study used in this research over various budget levels, that is to provide a fair basis for a comparative analysis. Given the special structure of the BD-BB, it has recently shown a significant computational superiority over other exact methods which makes it a valid and challenging yardstick to evaluate the proposed OA algorithm. The BD-BB comprises of solving some TAPs as well as a Benders decomposition problem which is a nonlinear optimization problem. The latter also includes solving a mixed integer programming problem as well as a capacitated traffic assignment problem which is computationally more expensive than a normal (uncapacitated) TAP, say more than four times [26]. To this end, Table 6 also reports on the number of times that a TAP and a Benders decomposition problem have been solved. As can be seen, a significant share of the CPU time has been occupied by the Benders decomposition. This has resulted in a higher CPU time needed to reach the global optimal solutions compared to what is needed by the OA (see column 4th and 9th of the Table). Note that, the OA fails to find the global optimal solution within 100 iterations for 2 out of 5 budgets, whereas, over the rest, the OA is remarkably faster. Moreover, as shown in Table 6, for the BD-BB, a significant share of the computation is attributed to the effort made to close the gap between a lower bound and an upper bound of the optimal value as a termination criterion (see column 9th versus column 8th of the Table). Consequently, the proposed OA can find a set of very good solution (and perhaps the global optimal solutions) in the very early iteration. This is what makes the OA appealing when a large-sized network is undertaken.

**Table 6:** Comparison to an exact method (Benders decomposition and branch and bound).

Global optimal solution		Outer Approximation of 100 iterations			Benders and Branch and bound [53]			
B/C%	Value of objective function	no of UE to reach a global optimal solution	CPU (min) to reach optimal solution	Total CPU (min)	no of UE solved*	no of Benders (lower bound) solved**	Total CPU (min)	CPU (min) to reach optimal solution
20	815035	53	8.05	14.93	4	160	60.6	36.97
40	808132	91	13.69	14.55	4	544	219	48.18
60	803900	Not found	NA	13.82	4	273	100.8	53.42
80	801692	Not found	NA	15	38	162	66	1.32
100	800928	13	1.72	12.37	41	55	24.6	12.55

*no of UE solved: number of times at which the traffic assignment is solved

**no of Benders (lower bound) solved that includes the number of times at which a pair of nonlinear programming problem (capacitated TAP, [58]) and mixed integer relaxed problem is solved

The closer a solution is to the optimal solution, the “better” it is. In the literature “good” solutions are defined in contrast with “exact” or “(global) optimal” solutions [14]. In general, it is not possible to measure the goodness of the solution since exact solutions are not known. In fact, the goodness of solution must be viewed as a qualitative and subjective rather than quantitative and decisive measure. We tried to show this concept in **Fig 5** where solutions are compared against intuitive solutions. Nonetheless, the y-axis in **Fig 5** indicates gap distance to corresponding exact solutions (found in exhaustive enumeration).

**Fig 6:**
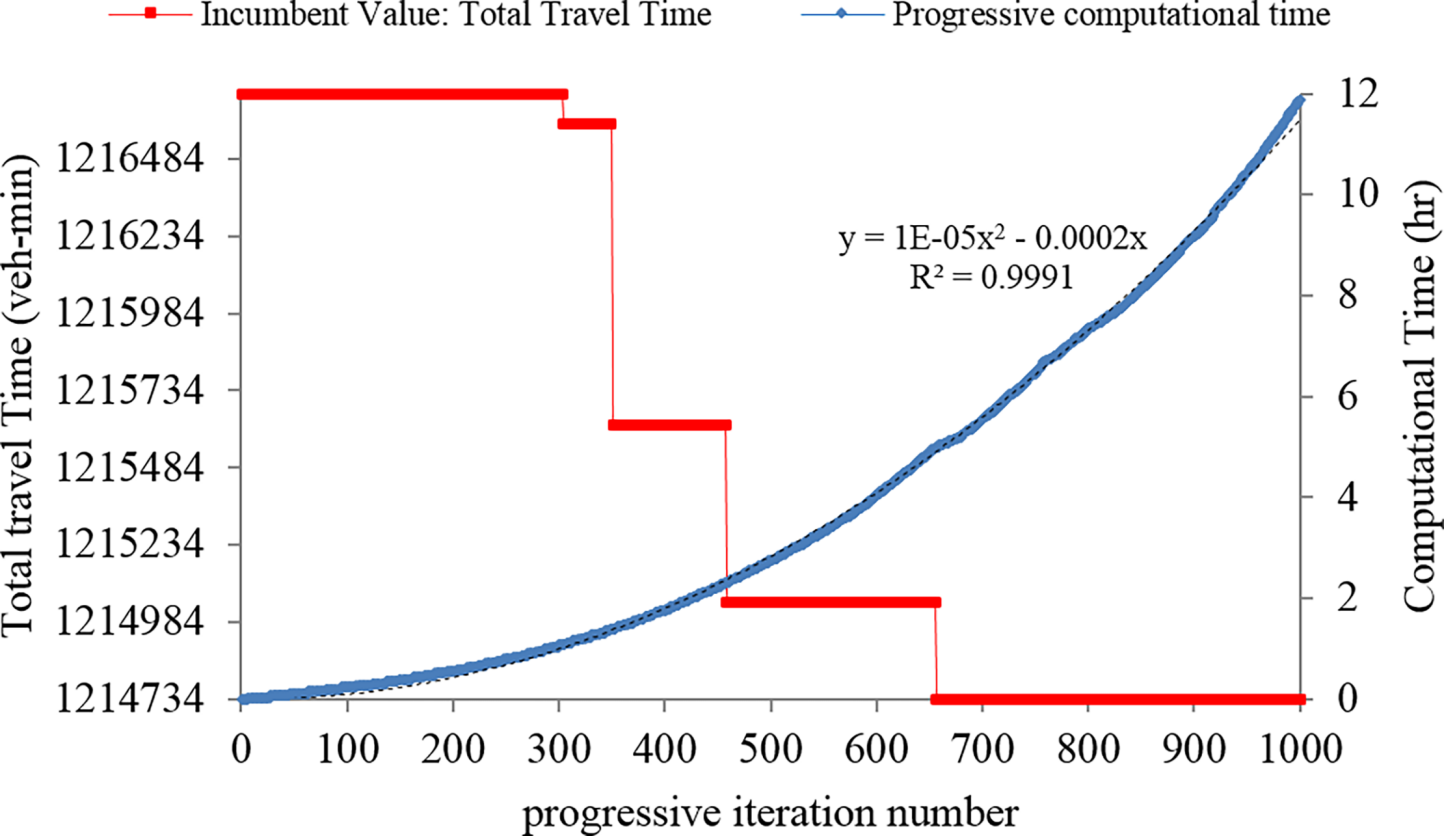
Winnipeg Case Study, variation of the results and computational burden for budget level B/C = 80%.

According to **Fig 5**, the algorithm does not yield a better solution than the intuitive solution for the budget level of B/C = 80% during the first 100 iterations. We left the algorithm to proceed until the optimal solution is found at iteration 657 and further until iteration 1000 as shown in **Fig 6** to observe the computational burden. The y-axis on the left-hand side shows the incumbent value (or objective function or total travel time). On the y-axis, the starting value (1214734) is the value of the objective function corresponding to the optimal solution. After 5 hr computational time, the optimal solution was found at iteration 657. The y-axis on the right-hand side shows the progressive computational time. As discussed earlier each iteration consists of solving a UE-TAP (which lasts almost 3 sec) and a MILP problem (DNDP-UE-MOA). As the number of iteration increases the number of cuts to the DNDP-UE-MOA increase (three cuts per iterations, see Table 1), hence the computational time increases too. Fortunately, the pace of such increase in the computational time is not exponential; rather it is of multinominal nature with the order of 2 (the trend line set on the computational curve is y = 1e-5x2-2e-4). Note that the authors don’t ascertain that this trend is always the case, hence more investigation on the growth of the CPU time is a worthy line of research. However, the main message is that as a hybrid methodology (exact and heuristic approximation), very good solutions are found in very early iterations as a promising sign when a large-sized network is undertaken.

Our methodology is similar to the method proposed by Wang, Meng (25) in the sense the objective function of the Beckmann transformation of UE traffic assignment is added to the constraints and an Outer Approximation algorithm is employed. Nevertheless, the main difference dwells on the fact that their method is basically a SO-relaxation formulation which postulates that SO-relaxation is a valid approximation to the original (UE) problem. Considering the theoretical gap between SO traffic flow and UE traffic flow (known as the price of anarchy) the more investigations using real-life examples is a worthy line of research.

## Conclusions

We developed a hybrid exact-heuristic method to address the Discrete Network Design Problem (DNDP) tailored to large-size networks in which, given a limited budget, the best subset of candidate road projects is sought. Conventionally, the DNDP is formulated as a bilevel programming problem: in the upper level, the network’s total cost is minimized while the lower level accounts for the commuters’ routing behavior. The decision variables are considered integer (binary): 1 to build and 0 not to build. Due to combinatorial nature and being NP-hard, the DNDP is known to be extremely difficult to deal with, for which we developed a two-tier relaxation scheme. First, the bilevel is relaxed to a single-level problem which is much easier and more efficient to handle. To this end, the User Equilibrium Traffic Assignment Problem (UE-TAP) in the lower level was considered as the main single-level problem. Then, the objective function in the upper level along with the binary constraints were included in the constraints of the single-level problem. As the result, a mixed-integer nonlinear programming problem is obtained (we referred to it as DNDP-UE-MINLP) for which an Outer Approximation (OA) solution algorithm was developed. Therefore, the DNDP was split to solving two problems alternately: (i) given a feasible binary solution, the corresponding UE-TAP is solved, (ii) given the outcome of the UE-TAP, the corresponding DNDP-UE-MINLP is linearized to a mixed-integer linear programming (MILP) and then solved using the OA. At this point, the outcome is a new binary solution. The second relaxation measure lays here: a fully-fledged linearization of the DNDP-UE-MINLP leads to a multi-commodity traffic flow, while the only takeaway is the binary solution, not the traffic flow. Hence we relaxed the multi-commodity formulation to a single-commodity. For the single-commodity, we merely retained the conservative flow constraints at the nodes which resulted in a MILP significantly trimmed off myriad variables and constraints. Nevertheless, to offset this simplification, the binary solution is used to fully solve the corresponding UE-TAP and the traffic flow. This process continues until a pre-specified number of iterations are exhausted. Furthermore, a good solution (optimal or near-optimal solutions) is always guaranteed in early iterations.

Note that, relaxing the multi-commodity to a single-commodity formulation to gain CPU efficiency comes at the expense of losing a tight lower bound. Therefore, a natural termination criterion is a maximum number of iteration (to be specified as a prior) is assumed to be a termination criterion which itself can be determined based on the computational technology of the time as well as the importance level of the problem. For example, if the total road investment projects is of the order of millions or billions dollars, it is conceivable to run the problem for weeks or even months to arrive at a better solution. Moreover, one intuitive way to set up a commensurate i_max (maximum number of iterations) is to observe the variations of the objective functions over successive results (see **Fig 4**). For instance, it is not recommended to terminate the computation when the graph fluctuates. Instead, one needs to leave the algorithm to carry on until the results remain unchanged for a considerable number of iterations.

The main challenge in the bilevel programming problems is the way to transform them into a single level problem. In the quest to arrive at a single-level problem, the consensus in the literature is to maintain the upper level since it is the leader in the original bilevel problem and then carry over the lower level (the follower) to the constraint. But, what we proposed was the other way around. In principle, by bringing the objective function down in the constraints and leaving Z (its upper bound) in the objective function, the structure of the formulation remains intact. However, the main difference is the relaxation method, that is, the multi-commodity formulation is replaced with a single-commodity formulation to cope with CPU burdens. Our primary motive was to tailor a working methodology for large-size networks (which is a rare currency in the literature). In the end; the numerical results showed a significant superiority over previous (conventional) methods. Furthermore, the way that we treated the leader objective function (by bringing it down to the constraints) has a significant advantage: it enables us to consider multiple objective functions for the DNDP. In addition to network performance index (e.x. minimizing total travel time), consideration of other concerns such as environmental factors, equity, traffic safety etc are sometimes strongly imperative [59, 60]. Such a situation gives rise to multi-objective optimization which itself is a challenging subject. Notably, the uniqueness of final solution in the multi-objective optimization might be compromised [61], not to mention the fact that the DNDP is per se an NP-hard problem.

The hybrid exact-heuristic algorithm was applied to four examples including Gao’s network and Sioux-Falls as well as the real network of the Winnipeg, Canada. Compared to the previous studies, the proposed algorithm showed significant superiority. Numerical testing on the real network of Winnipeg over various budget levels demonstrated promising results, such that the optimum solutions were quickly reached in most cases.

It is important to note that the DNDP is a NP-hard problem, that is, exact methods fail to solve it for the large-sized networks. Therefore, we developed a hybrid exact-heuristic consisting of an exact method and some heuristic relaxation techniques aiming to solve real-life networks. In terms of computational efficiency, we have depicted the CPU time that turned out to be a quadratic curve (not an exponential curve). However, in comparison to an all-out exact method (i.e. Benders decomposition and Branch and Bound, BB) our proposed method can find good (and sometimes the best) solution in very early iterations which is a promising sign to be used in real-life examples.

It is also important to highlight the fact the CPU time is a subjective factor considering the strategic level of the problems. Note that, theoretically, one cannot find the global optimal solutions for a large-sized example (NP-hard problem). That is why we let the number of iterations to be decided case-by-case depending on the computational technology at the time as well as the strategic level of the road investment. When millions or billions of dollars are at stake, it is conceivable to run the methodology for days and even months to find a better solution. However, one intuitive way to set up a commensurate i_max (maximum number of iterations) is to observe the variations of the objective functions over successive results (see **Fig 5**), to terminate the computation when no significant fluctuation is observed for some successive iterations. In other words, one needs to leave the algorithm to carry on until the results remain unchanged for a considerable number of iterations.

In a comparative context, the primary contribution of this research can be attributed to high computational efficiency and its potential application in real-life and large-size networks. Furthermore, the proposed formulation has the capacity to address multi-objective (or multi-criteria) network design problem. Second, by virtue of combinatorial nature of the problem, the discreteness of solutions may be compromised especially in large-size networks. Nonetheless, algorithms are required to be able to tightly minimize the objective function to ensure arriving at good solutions (i.e. solutions very close to the optimal solution). As shown in the numerical evaluation, the proposed algorithm can reach good solutions in early iterations.

The methodology proposed in this work could be further improved by expanding the traffic assignment to multi-class and multi-modal traffic flow. To enhance realism and fidelity of the traffic assignment models, the idea of subjecting the DNDP to a parking search model [62, 63] as well as dynamic network design assignment is of highest practical relevance that paves the way to use some off-the-shelf simulation software (the computational expense is, however, a serious concern [6, 64, 65]). In light of the scarcity of resources and cash flow issues, prioritization of selected projects out of solving the DNDP is also a demanding problem in the industry which deserves to be addressed [66]. Sometimes stakeholders seek a variety of good solutions other than a global optimum solution for reasons such as practicality issues, construction difficulties, and other concerns based on their own discretion or consensus. As such, similar to K-shortest-path problem finding the first k best solutions for the DNDP is worthy of further research. The DNDP has recently been expanded to more disaggregated levels in decision variables, such as finding additional lanes that need to be integrated into the proposed methodology. Mutual interactions of land use and road infrastructure are largely overlooked in the literature [67] which itself is a worthy line of research. Given the emerging technologies related to the autonomous and connected vehicles [68], it is important to investigate retrofitting existing transport and road infrastructure to accommodate these new modes.

## Appendix A: Outer approximation algorithm for MINLP

The general formulation of MINLP problems is [42] p.382:

MINLP:minf(x,y) (A-1)

s.t.g(x,y)≤0, (A-2)

$$x\in X\subseteq R^{n},y\in Y\subseteq Z^{m}$$ (A-3)

where *X* is a nonempty convex set in *R*^*n*^ (continuous variables) and *Y* is a finite integer set in *Z*^*m*^ (in case of binary variable we have *Y* = {1,0}^*m*^; *f*,*g* are convex in the space of (*x*,*y*). Consider MINLP, and let *S* be the solution space and *V* feasible (integer) decision solutions as:

S={(x,y)∈X×Y|g(x,y)≤0} (A-4)

V={y∈Y|∃x∈X,g(x,y)≤0} (A-5)

The OA alternates between solving a nonlinear programming subproblem and a mixed-integer linear programming master problem: The algorithm starts with *y*^*i*^ a feasible solution for decision variables at iteration *i* = 1. With this value of y fixed we consider solving the MINLP becomes a Non-Linear Programming (NLP) problem as follows:

$$NLP(y^{i}):\;v=\min\;f(x,y^{i})$$ (A-6)

$$s.t.\;g(x,y^{i})\leq 0,$$ (A-7)

$$x\in X\subseteq R^{n}$$ (A-8)

The *NLP*(*y*^*i*^) is solved and renders the continuous variables *x*^*i*^. Given the newly found *x*^*i*^, we proceed to find a new set of decision variable for the next iteration. Let us relax the *NLP*(*y*) problem from the constraints using Lagrangian multipliers *λ* ≥ 0: *d*_*y*_(*λ*) = min_*x*∈*X*_ *L*(*x*,*y*,*λ*) = *f*(*x*,*y*) + *λ*.*g*(*x*,*y*). Then the Lagrangian dual problem of *NLP*(*y*) becomes: max_*λ*_ *d*_*y*_(*λ*). Furthermore, since a solution of *NLP*(*y*) is also a feasible solution to *MINLP*, its optimal value *v*(*NLP*(*y*)) yields an upper bound to MINLP, therefore:

$$\underset{(x,y)\in S}{\min}f(x,y)\;=\min_{y\in V}\;v(NLP\;(y))=\min_{y\in V}(\max_{\lambda }\;\min_{x}\;L(x,y,\lambda ))$$ (A-9)

$$=\min\;z\;s.t.\;z\geq \min_{x}\;L(x,y,\lambda ),\;y\in V$$ (A-10)

where *z* is a lower bound to the optimal value of the original MINLP problem. The only constraint is a set of cuts to the solution space which is gradually accumulated during the progressive iterations. In doing so, the OA exploits the gradient property of the problem, both the objective function and the constraints. In fact, the objective functions and the constraints are represented by their linear approximation to form a Master Outer Approximation (MOA) as follows (note that the following equations can be derived directly due to the fact that the KKT conditions of *NLP*(*y*^*k*^) and its linearization at *x*^*k*^ are identical see (p. 383, c.13, [42]):

$$\begin{array}{l} MOA^{i}\;\min\;z\\ s.t.\;z\geq f(x^{k},y^{k})+\nabla f(x^{k},y^{k}).(x-x^{k},y-y^{k})\;k\in T^{i} \end{array}$$ (A-11)

$$0\geq g(x^{k},y^{k})+\nabla g(x^{k},y^{k}).(x-x^{k},y-y^{k})\;k\in T^{i}$$ (A-12)

x∈X,y∈Y (A-13)

The *MOA*^*i*^ is a mixed-integer linear programming (MILP) problem and *T*^*i*^ represent set of the solution *x*^*k*^, *y*^*k*^ found up to the current iteration *i*, in other words: *T*^*i*^ = {*k*|*y*^*k*^ ∈ *V and x*^*k*^ *solves NLP*(*y*^*k*^), *k* = 1..*i*}

It is worth noting that, constraint (A-12) represents all requirements to ensure that the outcome *y*^*i*+1^ is a feasible binary solution for the next iteration. Now the OA solution algorithm can be set forth as follow:

**Step 0.** Initialization: Set iteration *i* = 1 and choose a feasible solution for decision variables *y*^*i*^ ∈ *Y*. Initialize lower bound and upper bound as *lb*^0^ = −∞, $ub_{*}^{0}=+\infty$.
**Step 1**. Calculate Upper bound: Solve *NLP*(*y*^*i*^) to obtain *x*^*i*^. Update the value of best solution found by setting $ub_{*}^{i}=\min\{ub_{*}^{i-1},ub_{}^{i}=f(x^{i},y^{i})\}$. Save it as best solution (x*,y*) if it was found the best solution so far (known as incumbent value).
**Step 2.** Calculate Lower bound: Given *x*^*i*^ solve the master problem *MOA*^*i*^ to obtain optimal solutions of *z*^*i*^,*x*^*i*+1^,*y*^*i*+1^.
**Step 3.** Termination: Set *lb*^*i*^ = *z*^*i*^, if *lb*^*i*^ ≥ *ub*^*i*^ stop and (x*,y*) is the optimal solution, otherwise set *i* ≔ *i* + 1 and go to Step 1.
